# Validity and reliability of assessing strength and balance improvements by videoconference in pre-frail and frail older adults

**DOI:** 10.1007/s40520-025-03268-1

**Published:** 2025-11-28

**Authors:** Oliver J. Perkin, Ian Ju Liang, Carly D. McKay, Polly McGuigan, Max J. Western

**Affiliations:** 1 Department for Health, University of Bath, Bath, UK; 2 Division of Population Health and Genomics, School of Medicine, University of Dundee, Dundee, UK; 3 Department of Community Health Sciences, Cumming School of Medicine, University of Calgary, Calgary, Canada

**Keywords:** Physical function, Digital health, Telemedicine, Telerehabilitation, Older people/Adults, Frailty.

## Abstract

**Background:**

Assessing older adults’ physical function via videoconferencing technology is acceptable and feasible, enabling researchers and practitioners to monitor mobility remotely. However, validity of remote assessment in pre-frail and frail older adults, and its ability to detect change with intervention, has yet to be established.

**Aim:**

To establish the validity of remote physical function assessment in pre-frail and frail older adults compared to in-person assessment, and its reliability in detecting improvements in physical function during a 12-week exercise training intervention.

**Methods:**

Participants aged ≥ 65 years identified as pre-frail or frail based on in-person Short Physical Performance Battery scores ≤ 8 completed remote and in-person assessment of the 5x sit-to-stand, 60-second sit-to-stand, and single leg standing balance four times over 12-weeks (*n* = 49 at baseline, 40 at follow-up), with one group improving physical function with homebased exercise. Intraclass Correlation Coefficient and Bland Altman plots were used to determine agreement between assessment settings and consistency across the range of observed scores respectively in both Exercise and Control groups over time.

**Results:**

Remote assessment of sit-to-stand tests had good-to-excellent agreement with in-person assessment, and balance tests had moderate-to-good agreement. In the Exercise group, absolute bias in sit-to-stand tests was observed in both remote and in-person assessments at 4, 8, and 12 weeks, though this was not statistically different to the Control group.

**Discussion:**

Remotely assessing physical function in pre-frail and frail older adults is promising when compared to in-person assessments; however, there may be a bias towards better test performance over time when assessed remotely compared to in-person.

**Conclusions:**

Monitoring change in physical function in pre-frail and frail older adults using remote assessment is useful but should consider potential bias in measurement outcomes. Larger and more specific studies are needed to conclusively demonstrate the validity of remote assessment compared to in-person assessment when dealing with pre-frail and frail older people.

**Supplementary Information:**

The online version contains supplementary material available at 10.1007/s40520-025-03268-1.

## Introduction

The COVID-19 pandemic brought an inevitable challenge for health researchers and healthcare professionals worldwide. For example, social distancing and safety precautions meant that traditional face-to-face studies with in-person assessments could often not be undertaken. In response, many researchers employed technology to adapt their study methodologies [1–3] and technology-based telehealth and virtual visits/consultations services became increasingly common for a range of patients, including older adults [4–6].

Physical strength and balance is imperative for maintaining functional independence, overall well-being and quality of life during ageing [7, 8]. As such, assessing physical function is particularly important for predicting negative health outcomes, tailoring interventions, and evaluating treatment in older adults with limited mobility or at risk of frailty [9, 10]. Evidence supports that remote assessment of physical function via videoconferencing technology is both acceptable to research participants and feasible to conduct, albeit low digital literacy in older adults remains an obstacle for scaling implementation [11–13]. Given the ageing population and increasing prevalence of falls and mobility limitations and their consequences [14] determining whether digital tools are reliable for observing physical function more readily is an important question.

There is growing use of remote assessment of physical function in older adults and clinical populations as an alternative to in-person assessment [15–19]. While previous studies provide preliminary evidence suggesting that remote assessments may have results comparable to traditional in-person assessments, there is a need for validation studies specifically designed to compare remote and in-person collections of the same outcome, in representative populations [20]. In particular, evidence is limited regarding remote functional assessments in pre-frail and frail older adults, who may be most in need of support or intervention, or frequent monitoring over time [20, 21].

Given that exercise interventions seek to improve physical function and balance, it is vital that we determine whether remote testing protocols for these outcomes can reliably assess change. Intuitively, intrinsic and extrinsic factors might have varied effects on remote assessment validity. Intrinsic factors might include intraindividual variability in functional test performance. Lower function is associated with greater variability in strength test performance in older adults [22], so as function improves with exercise training, consistency in test performance may also improve (irrespective of test setting). Extrinsic factors might include physical characteristics in the assessment environment, such as carpet in the home versus hard floors in a laboratory impacting proprioception and balance test performance [23], or chair height impacting range of motion required for sit-to-stand performance [24]. These extrinsic factors might lead to greater differences in remote versus in-person assessment performance, thereby undermining remote assessment reliability. Other extrinsic factors such as participant motivation or alertness being affected by the process of travelling to an assessment centre or being observed in-person may also impact the outcomes of remote assessment compared to in-person assessment [25].

We previously reported a randomised controlled trial (RCT) evaluating the effectiveness of a homebased strength exercise snacking and tai-chi snacking intervention in pre-frail and frail older adults [26]. In a subgroup of participants, physical function was assessed both in-person and remotely on 4 occasions over 12-weeks. In these participants, the intervention produced clinically meaningful improvement in function based on the Short Physical Performance Battery (SPPB), moving many participants out of the pre-frail classification by the end of the intervention [27, 28]. The present study is a secondary analysis of the data collected in the RCT, which we use to evaluate the validity of remote versus in-person assessment of physical function in these pre-frail and frail older adults using videoconferencing technology in their own homes. Secondarily we examine the reliability of remote assessment for detecting improvements in physical function over the course of the 12-week intervention when compared to in person assessment.

## Methods

### Overview

This study involved secondary analysis of RCT data [26]. In the primary study, participants were randomly assigned to either twice daily strength and tai-chi ‘exercise snacking’, or to continue with their habitual activity patterns for 12 weeks (Exercise and Control groups respectively). Assessments of physical function were made via videoconferencing at 4-weekly intervals for all participants, with the first assessment at baseline prior to random group allocation. A subset of participants underwent additional in-person laboratory-based assessments of the same functional tests within the 7 days after the online assessment for each timepoint. At baseline, *n* = 49 participants completed assessment in both settings (Control = 22 vs. Exercise = 27), dropping to *n* = 42 at week 4 (Control = 20 vs. Exercise = 22), and *n* = 40 at weeks 8 and 12 (Control = 19 vs. Exercise = 21). The reason for the attrition across the study was business (*n* = 4), health or personal reasons (*n* = 1 each) in intervention participants, and dislike at not being in the experimental arm (*n* = 3) for control participants. Both videoconference and in-person assessments were conducted by the same researcher (IJL). Validity of remote versus in-person assessment in frail and pre-frail older adults was considered in all participants prior to the exercise intervention. The impact of improving physical function on remote testing outcomes was assessed by comparing groups at the subsequent timepoints. Ethical approval for the primary study was provided by the University of Bath Ethics Approval Committee for Health (REACH reference number: EP 20/21 082), and the trial registered on ClinicalTrials.gov (Identifier: NCT05758727).

### Participants

Full details regarding participant eligibility criteria for the primary RCT are available elsewhere [26]. Briefly, participants were eligible if they were aged 65 years or older, classed as pre-frail or frail based on scoring ≤ 8 in the full SPPB tested in person [29] (without scoring zero in any individual test component), and were not regularly engaging in exercise once a week or more. Written informed consent was provided online prior to undergoing screening.

### Videoconference assessments

Participants engaged in an online familiarisation session via video call, using participants’ preferred software (e.g., Zoom, Teams, FaceTime, WhatsApp), to become accustomed to the physical function tests and undergo an exercise safety screening assessment.

The remote assessments followed a previously established, feasible, and safe protocol [30]. Physical function was assessed with a timed 5 x sit-to-stands (5xSTS), a 60-second sit-to-stand test (60sSTS), and single-leg standing balance holds for a maximum of 60 s for both left and right legs. In the primary RCT, the SPPB balance tests were also performed. As balance test data are time-capped at 10 s across three stages of difficulty, analysing validity with Intraclass Correlation Coefficient (ICC) is not appropriate in this context.

For all functional tests, participants were asked to position their device (laptop, tablet, or mobile) such that their whole-body including feet could be seen by the researcher on the screen. The researcher provided verbal instructions to start and stop the tests, whilst timing manually with a stopwatch. The sit-to-stand tests were performed on a hard backed kitchen chair, and the participant was asked to use the same chair for each testing session. The 5xSTS was performed first and followed by the 60sSTS, and then single leg balance tests. Before each balance test, participants were asked to confirm that they felt safe to proceed. They were instructed to position themselves beside a stable surface (e.g., a table, kitchen counter, or sturdy chair) that could be used for support if required. Each test commenced with participants holding the surface, and timing began once they released their hands. No adverse events occurred during any of the assessments. Assessments were scheduled for approximately the same time of day for each repeated assessment. On the days that assessments were taking place, participants in the exercise group were requested not to perform their daily exercise prior to assessment. No other controls were imposed prior to testing, for example, refraining for caffeine intake.

### In-person assessments

Participants were invited to the Human Physiology Laboratory at the University of Bath for in-person evaluations of the same assessments undertaken remotely via videoconferencing. The laboratory-based assessments replicated the order of tests procedures previously conducted during the videoconference familiarisation. Thereafter, the timed 4-meter walk component of the SPPB and the timed-up-and-go (TUG) [31] tests were completed to characterise participants functional capacity against recognised criteria at each timepoint.

### Statistical analysis

Intraclass Correlation Coefficient (see equation) with 95% confidence intervals [95%CI] was used to assess criterion validity of remote assessment compared to in-person assessment within all participants at baseline (whilst still pre-frail/frail) [32, 33]. Specifically, the ICC two-way mixed-effects model was employed, considering the absolute agreement between single measurements [34].

$$\:\frac{{\text{M}\text{S}}_{\text{R}}\:-\:{\text{M}\text{S}}_{\text{E}}}{{\text{M}\text{S}}_{\text{R}}\:+\:(k-1){\text{M}\text{S}}_{\text{E}}+\frac{k}{n}({\text{M}\text{S}}_{C}-{\text{M}\text{S}}_{\text{E}})}$$

Notes; MS_R_ = mean square for rows; MS_E_ = mean square for error; MS_*C*_ = mean square for columns; *n* = number of subjects; *k* = number of raters/measurements.

Less than 0.50 was taken to indicate ‘’Poor agreement’’; 0.50 to 0.75 to indicate ‘’Moderate agreement’’; 0.75 to 0.90 to indicate ‘’Good agreement’’; and over 0.9 to indicate ‘’Excellent agreement’’ [35]. To compare reliability of remote testing between groups at a given timepoint after the baseline assessment, 95% confidence intervals of the ICCs were inspected. Where there was no between group overlap of 95% confidence intervals for ICCs between assessment settings, the validity was assumed to be different between groups [33].

Bland-Altman plots present agreement between remote and in-person assessment settings for each group at each timepoint [36, 37]. Within group absolute bias for each timepoint was determined if the line of equality was outside the confidence interval of the mean difference. Non-overlapping 95% confidence intervals of the mean difference between Bland-Altman plots for each group at a given timepoint signalled a difference in bias between groups. Proportional bias was assessed by linear regression (difference between assessment settings-by-mean of assessments settings), with a mean-by-group interaction term to assess whether the proportional bias differed between groups at a given timepoint [38]. As the original RCT was not powered for this secondary analysis, overlapping confidence intervals was used as a cautious approach to establish differences in agreement between conditions that were robust and unlikely to be due to chance [39]. All statistical analyses were performed using RStudio 2024.04.2 Build 764 “Chocolate Cosmos” for Windows, R version 4.1.2 (R Core Team 2021, R Foundation for Statistical Computing, Vienna, Austria). Statistical significance was accepted at *p* < 0.05.

## Results

### Participants

Participant characteristics are presented in Table 1, including SPPB score, and TUG time recorded at each timepoint during in-person testing. The intervention significantly improved functional status with clinically meaningful improvements in SPPB observed in the Exercise group compared to the Control group by linear mixed effects modelling [26].

**Table 1:** Participant characteristics

		Control group	Exercise group	
Age (years)		75 ± 5	74 ± 6	
Height (cm)		169.5 ± 8.9	169.1 ± 8.5	
Body mass (kg)		75.7 ± 12.8	72.0 ± 11.8	
*n* (% females)	*Baseline*	22 (59%)	27 (67%)	
	*Week 4*	20 (65%)	22 (82%)	
	*Week 8*	19 (68%)	21 (86%)	
	*Week 12*	19 (68%)	21 (86%)	
Functional characteristics				*p value*
SPPB score /12	*Baseline*	7.8 ± 0.6	7.7 ± 0.7	-
	*Week 4*	8.0 ± 2.3	10.2 ± 1.4	*< 0.001*
	*Week 8*	8.8 ± 1.0	10.7 ± 1.6	*< 0.001*
	*Week 12*	8.9 ± 1.0	11.2 ± 1.2	*< 0.001*
TUG (seconds)	*Baseline*	11.1 ± 2.0	10.7 ± 2.7	-
	*Week 4*	11.7 ± 2.1	10.0 ± 2.4	*0.002*
	*Week 8*	10.5 ± 2.1	*9.6* ± 2.4	*0.008*
	*Week 12*	10.5 ± 2.1	*8.8* ± *1.7*	*0.071*

Note: data presented as mean ± standard deviation unless otherwise state. Abbreviations: *n*; number of participants measured at each timepoint, SPPB; short physical performance battery, TUG; timed-up-and-go test. *p* values represent between group difference analysed over time using linear mixed models adjusted by sex and age for all follow-up measures [26]: *Liang*,* I.J.*, et al.,* The Efficacy of 12-Week Progressive Home-Based Strength and Tai-Chi Exercise Snacking in Older Adults: A Mixed-Method Exploratory Randomised Control Trial. The Journal of Frailty & Aging*,* 2024*

### Validity of remote versus in-person functional testing and reliability with improvements in physical function

For all participants at baseline, the validity of the remote sit-to-stand assessments (5xSTS and 60sSTS) was high, with good-to-excellent agreement with in-person assessments (ICC [95% CI] of 0.84 [0.74–0.91] and 0.82 [0.71–0.90] respectively (Table 2). For the single leg balance tests on both right and left legs assessed remotely compared to in-person, ICCs indicated moderate-to-good agreement (0.75 [0.59–0.85] and 0.78 [0.64–0.87] respectively, Table 2).

The reliability of remote compared to in-person assessment did not change as functional status improved during the exercise intervention. The 95% confidence intervals of the ICC between remote and in-person assessment scores for the Exercise group overlapped with those of the Control group at all timepoints after baseline assessment (Table 2).

**Table 2:** Intraclass correlation coefficients between in-person and remote assessment scores of functional tests by group

Functional test performed in all pre-frail/frail participants at baseline	Assessment setting		Validity				
	In-person	Remote	ICC [95% CI]	Agreement			
5xSTS (seconds)	16.5 ± 4.1	16.6 ± 5.0	0.84 [0.74–0.91]	Good to excellent			
60sSTS (reps)	21 ± 6	21 ± 8	0.82 [0.71–0.90]	Good to excellent			
Right leg standing balance (seconds)	25.9 ± 24.4	24.6 ± 21.7	0.75 [0.59–0.85]	Moderate to good			
Left leg standing balance (seconds)	26.3 ± 21.9	25.9 ± 21.9	0.78 [0.64–0.87]	Moderate to good			

Functional test	Timepoint	Control group			Exercise group		
		In-person	Remote	ICC [95% CI]	In-person	Remote	ICC [95% CI]
5xSTS (seconds)	*Week 4*	15.3 ± 3.8	15.5 ± 4.4	0.92 [0.82–0.97]	13.0 ± 3.3	12.4 ± 3.3	0.94 [0.82–0.98]
	*Week 8*	14.2 ± 3.3	14.4 ± 3.6	0.90 [0.76–0.96]	12.3 ± 4.4	11.8 ± 4.0	0.92 [0.81–0.97]
	*Week 12*	14.1 ± 3.6	13.8 ± 2.7	0.85 [0.66–0.94]	11.5 ± 3.4	10.6 ± 3.5	0.93 [0.57–0.98]
60sSTS (reps)	*Week 4*	21 ± 7	22 ± 7	0.86 [0.67–0.94]	23 ± 6	26 ± 7	0.76 [0.30–0.91]
	*Week 8*	22 ± 7	23 ± 6	0.81 [0.58–0.92]	25 ± 7	28 ± 9	0.80 [0.44–0.93]
	*Week 12*	22 ± 6	25 ± 7	0.70 [0.35–0.87]	26 ± 6	30 ± 9	0.78 [0.11–0.93]
Right leg standing balance (seconds)	*Week 4*	15.9 ± 16.7	21.5 ± 19.5	0.67 [ 0.34–0.84]	31.7 ± 22.3	32.6 ± 22.1	0.87 [ 0.71–0.94]
	*Week 8*	18.7 ± 17.3	23.6 ± 18.6	0.73 [0.43–0.89]	34.0 ± 24.1	33.3 ± 23.5	0.95 [0.87–0.98]
	*Week 12*	16.9 ± 14.4	26.0 ± 21.2	0.49 [0.08–0.76]	34.6 ± 23.7	38.3 ± 22.9	0.80 [0.57–0.91]
Left leg standing balance (seconds)	*Week 4*	23.8 ± 20.0	16.0 ± 16.4	0.46 [0.06–0.74]	34.3 ± 23.2	35.7 ± 21.6	0.67 [0.34–0.85]
	*Week 8*	23.2 ± 22.1	22.7 ± 17.7	0.70 [0.37–0.88]	35.1 ± 23.2	36.0 ± 22.5	0.81 [0.59–0.92]
	*Week 12*	18.7 ± 17.3	22.6 ± 18.4	0.68 [0.35–0.86]	33.4 ± 21.3	34.2 ± 22.2	0.79 [0.57–0.91]

Notes: Data are presented as mean ± SD. No between group differences were identified in ICC of in-person and remote assessments at any timepoint. Abbreviations- 5xSTS; time taken to perform five repeated sit-to-stands at maximum speed, 60sSTS; number of complete sit-to-stand repetitions performed in 60 s

### Agreement between remote and in-person functional testing in pre-frail and frail older adults

**Fig. 1:**
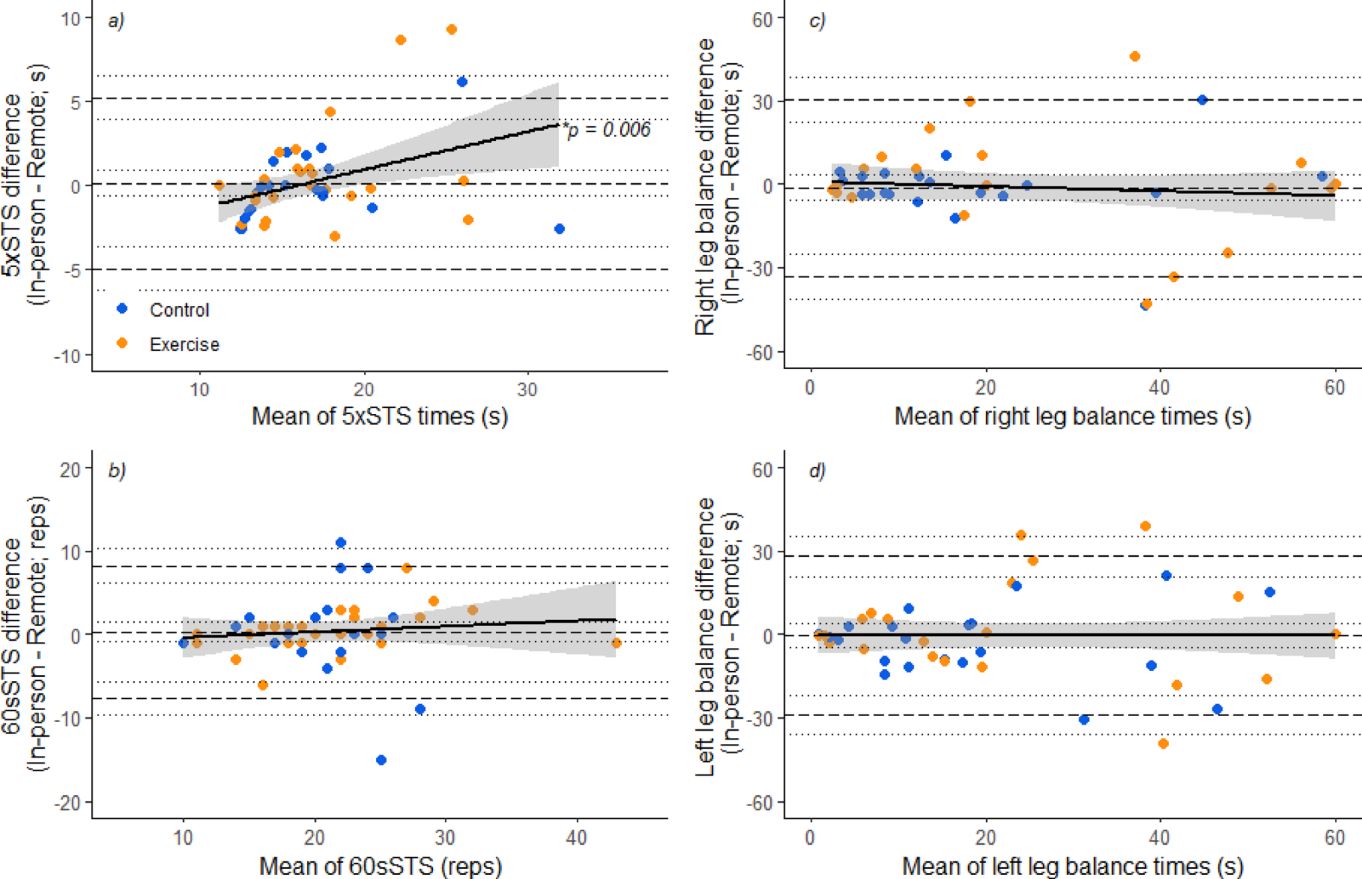
Bland-Altman plots for 5 x sit-to-stand tests (**a**), 60-second sit-to-stand tests (**b**), and right and left leg single balance (**c** & **d**) respectively assessments performed remotely and in-person, with 95% confidence intervals around lines of mean difference, and upper and lower limits of agreement. Positive differences (y-axis) indicate repetitions were performed more quickly in the in-person assessment than the remote assessment the 5 x sit-to-stand test (**a**), that more repetitions were performed in the remote assessment than the in-person assessment for the 60-second sit-to-stand tests (**b**), or longer balance hold durations were performed in the in-person assessment than the remote assessment (**c** & **d**). Linear regression with 95% confidence intervals is presented in black lines, with *p* values next to linear regression denoting significant proportional bias. Abbreivations: 5xSTS; 5 x sit-to-stand test, 60sSTS; 60-second sit-to-stand test, reps; repetitions

Agreement was addressed in terms of absolute and proportional bias using Bland-Altman plots and linear regression. There was no evidence of absolute bias in neither sit-to-stand nor single leg balance tests assessed remotely when compared to in-person (Fig. 1). In the 5xSTS assessment, proportional bias was observed (R^2^ = 0.23, *p =* 0.006), i.e., participants who performed worse in the 5xSTS overall were likely to perform the assessment more slowly when assessed in-person compared to remotely. Proportional bias between assessment conditions was not observed in the 60sSTS or single leg balance tests.

### Agreement between remote and in-person functional testing with improved in physical function

**Fig. 2:**
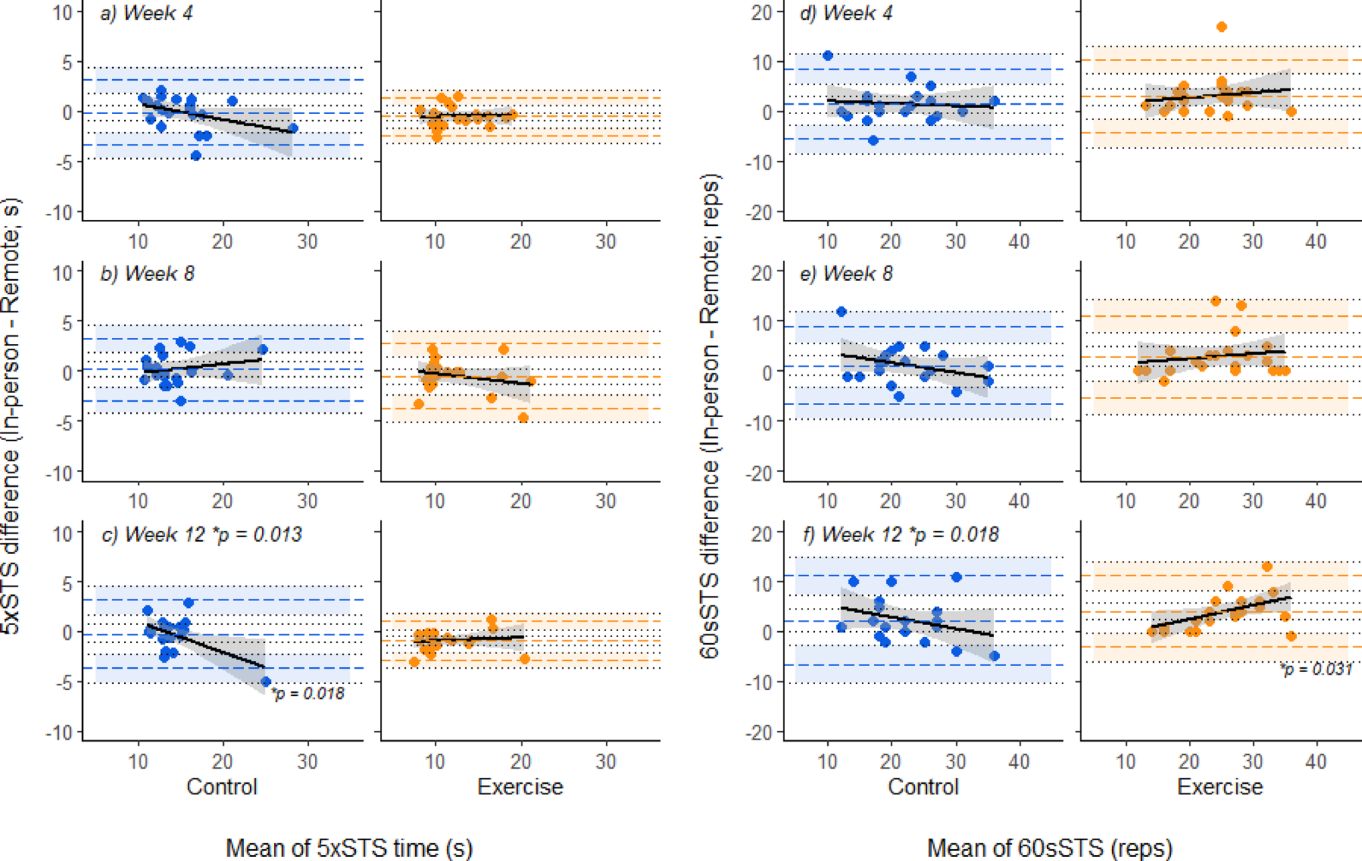
Bland-Altman plots for 5 x sit-to-stand tests (**a**-**c**) and 60-second sit-to-stand tests (**d**-**e**), paired by timepoint, with 95% confidence intervals around lines of mean difference, and upper and lower limits of agreement (the latter shaded for ease of comparison between groups at a given timepoint). Positive differences (y-axis) indicate repetitions were performed more quickly in the in-person assessment than the in remote assessment the 5 x sit-to-stand test (**a**-**c**), or that more repetitions were performed in the remote assessment than the in-person assessment for the 60-second sit-to-stand tests (**d**-**e**). Linear regression with 95% confidence intervals is presented in black lines, with *p* values next to linear regression denoting significant proportional bias, and *p* values next to figure panel labels denoting significant difference in proportional bias between groups at a given timepoint. Abbreviations: 5xSTS; 5 x sit-to-stand test, 60sSTS; 60-second sit-to-stand test, reps; repetitions

In the 5xSTS, absolute bias was observed in the Exercise group at weeks 4 and 12, with the test completed more quickly in the remote assessment than the in-person assessment by 0.6 [0.1–1.0] seconds and 0.9 [0.5–1.4] seconds respectively (Figs. 2a and b). However, there were no statistical between group differences in absolute bias for any given timepoint, i.e., 95% confidence intervals of line of equality overlapped between groups at all timepoints.

In the 60sSTS, there was absolute bias in Exercise group at weeks 4 and 8 indicating more repetitions were performed in the remote assessment than in the in-person assessment (2.9 [1.3–4.6] and 2.9 [1.0–4.8] repetitions more respectively (Figs. 2d and e)). At 12 weeks, absolute bias (more repetitions completed in the remote compared to the in-person assessment) was seen in both groups, by 4 [2.3–5.7] repetition in the Exercise group, and 2.3 [0.1–4.4] repetitions in the Control group (Fig. 2f). Absolute bias was not statistically different between groups at any timepoint.

Proportional bias was observed in the Control group at week 12 in the 5xSTS test, with slower mean 5xSTS times associated tendency for faster times at in-person assessment compared to remote assessment (R^2^ = -0.31, *p* = 0.018), and a difference in proportional bias between groups was also detected at week 12 (*p* = 0.013; Fig. 2c). Proportional bias was evident in the Exercise group’s 60sSTS test performance at 12 week (R^2^ = 0.27, *p* = 0.031) and different between groups (*p* = 0.018; Fig. 2f).

**Fig. 3:**
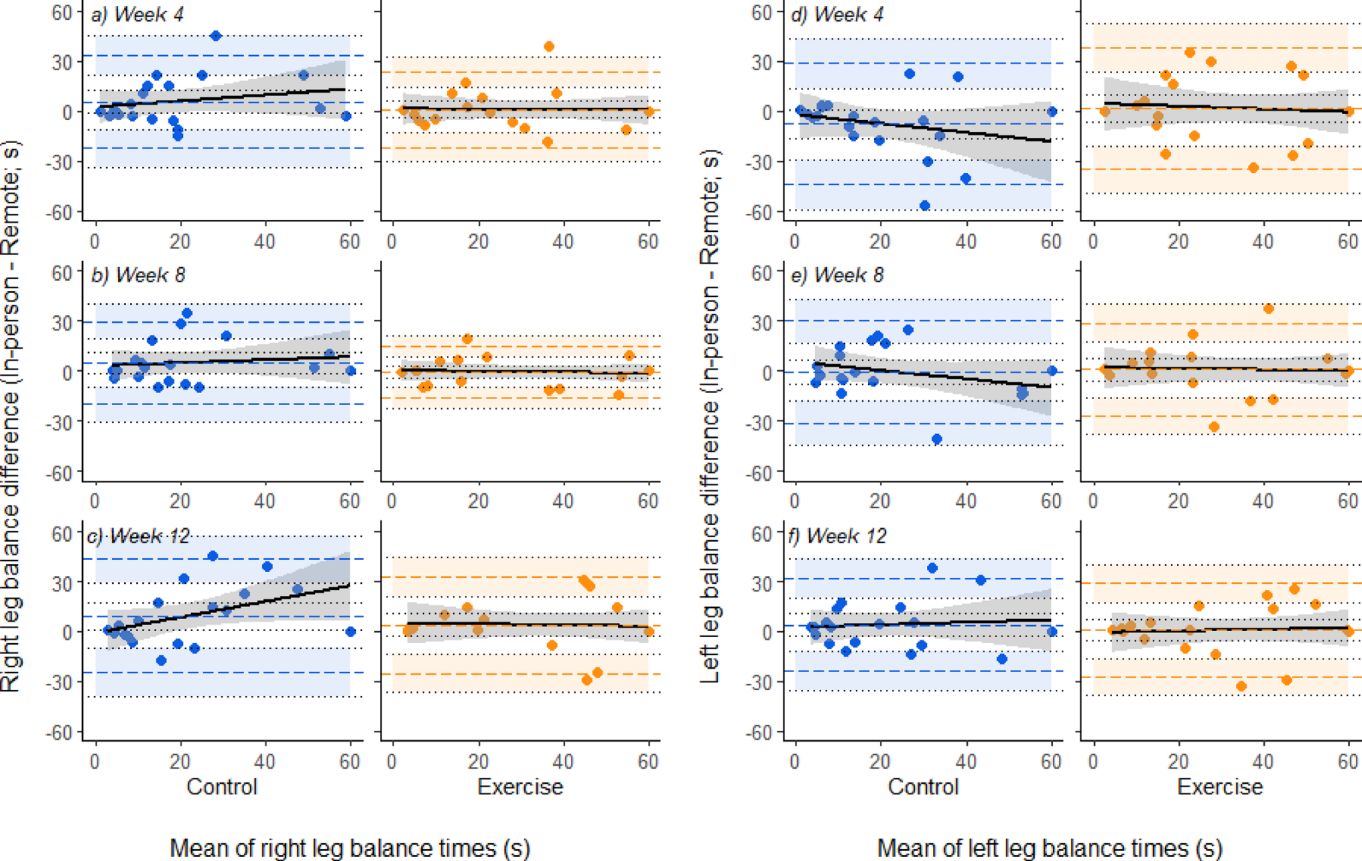
Bland-Altman plots for right leg (**a-d**) and left leg (**e-h**) unilateral standing tests, paired by timepoint, with 95% confidence intervals around lines of mean difference, and upper and lower limits of agreement (the latter shaded for ease of comparison between groups at a given timepoint). Positive differences (y-axis) indicate longer balance hold durations were performed in the in-person assessment than the remote assessment. Linear regression with 95% confidence intervals is presented in black lines

In the single leg balance tests, there was absolute bias observed in the Control group performance at 12 weeks, with the right leg balance hold performance being 9.2 [0.8–17.5] seconds better in the in-person assessment condition (Fig. 3c). This was not statistically different to the Exercise group, and there was no indication of absolute or proportional bias at any other single leg balance test timepoint in either group.

## Discussion

The present study demonstrates that remote assessment of two common sit-to-stand tests is a promising option for measuring physical function in pre-frail and frail older adults when compared to in-person assessment. After 4 weeks of homebased exercise that improved physical function, there was evidence of better sit-to-stand performance in the remote assessment than the in-person assessment in the Exercise group. However, this absolute bias was not statistically different from the Control group, who themselves performed more sit-to-stand repetitions in 60 s in the remote compared to in-person assessment at 12 weeks. Remote single leg balance assessments had moderate-to-good agreement with in-person assessment for pre-frail and frail older adults, and improving physical function did not lead to differences in the reliability of remote assessment between groups.

The finding that 5xSTS and 60sSTS assessments performed remotely agree well with in-person assessment for pre-frail and frail older adults is encouraging, given that many studies are adopting remote assessment methods in older adults [20, 21]. The present ICC scores generally agree with previous literature in non-frail older adults in this respect, but may be considered to be on the lower end of the typically observed range [21]. In non-frail older adults, ICC between remote versus in-person assessment of 5xSTS has been reported as 0.96 [0.89–0.99] and 0.94 [0.88–0.97] for the 30 s sit-to-stand [19, 40]. The ICCs between remote and in-person assessment were 0.84 [0.74–0.91] and 0.82 [0.71–0.90] for the 5xSTS and 60sSTS respectively in the present study. Notably, previous authors speculated that agreement would be different in lower functioning older adults [40]. This certainly appeared to be the case in the single leg balance assessments. Whilst in non-frail older adults ICC for single leg balance testing was 0.94 [0.89–0.97] (good-to-excellent), agreement in the present study was lower at 0.75 [0.59–0.85] and 0.78 [0.64–0.87] for right and left leg balance, respectively. This may be indicative of balance being a less stable marker of physical function in more frail older persons, and impacted by external factors such as mental or physical fatigue, prior activity, hydration and time of day [41, 42]. Moreover, the single leg balance performance generally appeared to be more variable compared to the sit-to-stand tests over time in the present study, irrespective of assessment setting.

The concept that functional status is an intrinsic factor impacting remote assessment validity is supported by the Bland-Altman statistics identifying proportional bias in the 5xSTS assessment in pre-frail and frail participants at baseline. The longer participants took to perform the 5xSTS, the larger the difference in performance between assessment settings (R^2^ = 0.23, *p* = 0.006; Fig. 1a). The trend was for better remote performance than in person performance and given that remote assessment was performed before the in-person assessment, this suggests that it was not due to familiarisation effects. Rather, this supports that for the lower functioning older adults, agreement between assessment conditions in sit-to-stand tests may be at greater risk of bias. However, in this context it should be noted that the 5xSTS is typically incorporated in the SPPB [27]. Given that the threshold for the score of 1 (the lowest completing score) in the SPPB is 16.7 to 60.0 s, it does not appear that this bias would change SPPB composite scores.

The results of this study suggest that that improvements in physical function following homebased exercise interventions may be systemically overestimated in sit-to-stand tests when assessed remotely compared to a laboratory setting. The Exercise group displayed absolute bias in the 5xSTS at 4 and 12 weeks, and in the 60sSTS at all timepoints during the intervention, based on Bland Altman plots [37] (Fig. 2). However, when comparing to the Control group, the 95% confidence intervals around the line of equality for the respective groups overlapped at all timepoints, indicating that the absolute bias was not different between groups. Indeed, at the 12-week timepoint, the Control group also displayed absolute bias towards improved performance in the remote assessment compared to the in-person assessment for the 60sSTS.

Several factors may contribute to a better performance in the sit-to-stand tests performed remotely compared to in the laboratory, particularly in the Exercise group. Whilst these could be considered as intrinsic and extrinsic factors, there are clear links and, in some instance, cross-over between potential sources of bias. For example, as an extrinsic factor, the chair used for remote assessment might have been higher, thus reducing range of motion required for a single repetition so making them easier to perform. However, if this were an influencing factor in and of itself, then it might be expected that this would not be different between the randomly allocated groups. Given the exercise snacking intervention featured sit-to-stands from a chair for 60 s, familiarity with the chair height might have contributed to a trend towards better performance in the remote condition in the Exercise group, much akin to the classic concept of specificity of training [43]. Interestingly, the right leg balance assessment for the Control group at 12-weeks showed absolute bias towards better performance in the in-person assessment by 9.2 [0.8–17.5] seconds. Again, this was not different between groups, and it is difficult to rationalise what caused this difference in performance between settings, given that extrinsic factors such as differences between floor type would have been the same at all timepoints and for both groups, and there were no obvious changes to intrinsic factors for the Control group.

There were 2 instances of proportional bias in the agreement between remote and in-person testing in the sit-to-stand tests that are noteworthy for different reasons. In the 5xSTS, there was proportional bias in the Control group at 12-weeks which would indicate that participants who took longer to perform 5 sit-to-stands tended to perform better in the remote assessment than in the in-person assessment (Fig. 2c). This proportional bias was statistically different to the Exercise group at 12 weeks. However, removing the observable outlier from the Control group as a sensitivity analysis eliminated the proportional bias (R^2^ = 0.08, *p* = 0.723) and difference in proportional bias between groups (*p* = 0.822). Conversely, in the 60sSTS at 12 weeks, there was a robust statistical difference in proportional bias between groups (Fig. 2f). Those in the Exercise group performing the most repetitions in the 60sSTS (i.e., highest functioning) actually displayed the greatest difference between assessment conditions. Fundamentally, these data somewhat undermine the concept that higher physical function per se would lead to more consistent physical function test performance, based on lower function being associated greater variability is strength test performance [22]. It might be more nuanced to consider that these data indicate the specificity of the daily exercise intervention that includes squatting to the sit-to-stand test [inevitably] improves performance in the test. In any case, whilst there were no differences in absolute bias between groups caused by improving physical function of pre-frail and frail older adults, studies employing homebased exercise that conduct their functional assessments entirely online should consider the possibility that improvements in function compared to control groups might be positively biased towards the homebased assessment setting.

A strength of the present study was having the same researcher conduct both remote and in-person assessments, allowing for isolated comparison of the assessment settings without issues of interrater reliability. Furthermore, the repeated measures design and inclusion of pre-frail and frail older adults specifically adds to the body of literature currently available on validity of remote assessment of physical function in older adults [20, 21]. However, there are some study limitations that should be acknowledged. Firstly, the timed walking tests were not considered feasible to assess remotely, owing to limited space within participants’ homes to perform the testing whilst in view of a screen, and the logistical challenges of delivering calibrated devices for measuring out walk length to participants. While the researcher administering both sets of assessments being the same across all trials mitigates against inter-rater variability, this approach increases the risk of observer bias given the rater’s awareness of prior performance and study group. Furthermore, the relatively small sample size reduces confidence in the ICC estimates due to greater variability, and risks an influence by outliers on the statistical tests (it is not uncommon for older adults with mobility limitations to not get adequately set in balance tests, for example) [44, 45]. Although the data do not suggest it was an issue, the order of remote and in-person assessments may also have been a source of bias, as might the period of time between trials which ranged from 0 to 7 days. It is possible that participants’ performance could have varied slightly over this period due to day-to-day fluctuations in function, fatigue, or motivation. A randomised, assessor blind, counterbalanced trial order would have been a preferable model to structure remote versus in-person assessments for examining validity of remote assessment. However, in the RCT from which these data were sourced, the primary outcome was remotely assessed physical function. Fidelity of those data was paramount, so remote assessment was undertaken first, and flexibility in in-person assessment scheduling was required for the feasibility of the RCT.

As mentioned, participants used available chairs in their homes for sit-to-stand tests, introducing a lack of control over equipment consistency in the 2 settings. While it is not possible to say exactly whether or how this influenced the remote assessment validity, this may actually improve generalisability of findings given few studies will provide chairs to participants for remote testing. Similarly, technical issues with videoconferencing connection, positioning of cameras, differences in floor type between the assessment settings and pre-test context (fatigue etc.) may all introduce possible bias but will likely be present in many remote assessment settings. These factors should remain consistent for a given individual in repeated measures studies, and robust randomisation and larger sample sizes should render their impact negligible.

Future research should seek to evaluate the validity of remote testing in the context of improving physical function with exercise interventions of longer duration and include a greater range of functional assessments [46]. The inclusion of multiple raters blinded to study group allocation and prior assessment scores would strengthen future studies, reducing risk of bias associated with a single assessor, and demonstrating scalability for studies with greater sample sizes. Furthermore, whilst the present study recruited participants based on having some degree of strength and balance mobility limitations, they were still required to be capable of homebased strength exercise and tai-chi snacking. Other prerequisites of participation were sufficient digital literacy to operate videoconferencing and having someone available in the house during remote assessment and daily exercise bouts [26]. Future research should seek to establish validity of remote physical function testing in older populations defined as frail using other metrics such as Fried Frailty Phenotype [47] or Clinical Frailty Score [48]. Similarly, the present findings may not generalise to cohorts of different socioeconomic status, ethnicity or culture, and participant inclusivity in future research into validity of remote assessment must be improved [49, 50]. A useful direction for research would be to firstly minimise extrinsic and intrinsic factors in testing the validity of remote assessment vs. in-person assessment and secondly identifying cutoffs for physical function tests where remote assessment can no longer be considered valid (and strategies to overcome this).

## Conclusion

The present findings support that remote assessment of sit-to-stand and single leg balance tests are comparable to in-person assessment in pre-frail and frail older adults. However, homebased exercise training that improves physical function may lead to bias in sit-to-stand test performance whereby participants do better when assessed remotely compared to in-person. Future research or practice evaluating change in function should consider risk of this bias overestimating the effect of interventions remotely.

## Supplementary Information

Below is the link to the electronic supplementary material.

Supplementary Material 1

## Data Availability

The data and analytic methods are available upon request from the first or corresponding authors.
