# Supplementary material for: Validity and reliability of assessing strength and balance improvements by videoconference in pre-frail and frail older adults
Source: Aging Clin Exp Res. 2025 Nov 28;38(1):15. doi: 10.1007/s40520-025-03268-1 (PMC12753708; doi:10.1007/s40520-025-03268-1)
Supplement: Supplementary file 1 — Supplementary Material 1 [file 40520_2025_3268_MOESM1_ESM.docx]

| **Functional test performed in all pre-frail/frail participants at baseline** | | | **Mean difference between assessment settings**  **(Remote – In-person) [95% CI]** | | | | **ICC [95% CI]** | |
| --- | --- | --- | --- | --- | --- | --- | --- | --- |
| **5xSTS (seconds)** | | | 0.2 [-1.7 – 2.0] | | | | 0.84 [0.74 – 0.91] | |
| **60sSTS (reps)** | | | 0 [-2 – 3] | | | | 0.82 [0.71 – 0.90] | |
| **Right leg standing balance (seconds)** | | | -1.3 [-10.4 – 7.7] | | | | 0.75 [0.59 – 0.85] | |
| **Left leg standing balance (seconds)** | | | -0.4 [-9.2 – 8.4] | | | | 0.78 [0.64 – 0.87] | |
| **Functional test** | **Timepoint** | **Control group** | | |  | **Exercise group** | | |
|  |  | **Mean difference**  **(Remote – In-person)**  **[95% CI]** | | **ICC [95% CI]** |  | **Mean difference**  **(Remote – In-person)**  **[95% CI]** | | **ICC [95% CI]** |
| **5xSTS (seconds)** | *Week 4* | 0.2 [-2.8 – 2.5] | | 0.92 [0.82 – 0.97] |  | -0.6 [-2.6 – 1.4] | | 0.94 [0.82 – 0.98] |
|  | *Week 8* | 0.2 [-2.1 – 2.5] | | 0.90 [0.76 – 0.96] |  | -0.5 [-3.1 – 2.1] | | 0.92 [0.81 – 0.97] |
|  | *Week 12* | -0.3 [-2.4 – 1.8] | | 0.85 [0.66 – 0.94] |  | -0.9 [-3.1 – 1.2] | | 0.93 [0.57 – 0.98] |
| **60sSTS (reps)** | *Week 4* | 1 [-3 – 6] | | 0.86 [0.67 – 0.94] |  | 3 [ -1 – 7] | | 0.76 [0.30 – 0.91] |
|  | *Week 8* | 1 [-3 – 5] | | 0.81 [0.58 – 0.92] |  | 3 [-2 – 8] | | 0.80 [0.44 – 0.93] |
|  | *Week 12* | 2 [-2 – 6] | | 0.70 [0.35 – 0.87] |  | 4 [-1 – 9] | | 0.78 [0.11 – 0.93] |
| **Right leg standing balance (seconds)** | *Week 4* | 5.6 [-6.0 – 17.2] | | 0.67 [ 0.34 – 0.84] |  | 0.9 [-12.7 – 14.4] | | 0.87 [ 0.71 – 0.94] |
|  | *Week 8* | 4.9 [-6.9 – 16.7] | | 0.73 [0.43 – 0.89] |  | -0.7 [-15.6 – 14.1] | | 0.95 [0.87 – 0.98] |
|  | *Week 12* | 9.1 [-2.8 – 21.1] | | 0.49 [0.08 – 0.76] |  | 3.8 [-10.8 – 18.3] | | 0.80 [0.57 – 0.91] |
| **Left leg standing balance (seconds)** | *Week 4* | -7.8 [-19.5 – 3.9] | | 0.46 [0.06 – 0.74] |  | 1.4 [-12.3 – 15.0] | | 0.67 [0.34 – 0.85] |
|  | *Week 8* | -0.5 [-13.7 – 12.6] | | 0.70 [0.37 – 0.88] |  | 0.9 [-13.4 – 15.1] | | 0.81 [0.59 – 0.92] |
|  | *Week 12* | 3.9 [-7.9 – 15.6] | | 0.68 [0.35 – 0.86] |  | 0.8 [-12.8 – 14.4] | | 0.79 [0.57 – 0.91] |

Supplementary Table 1. Mean difference in functional test performance and intraclass correlation coefficients between in-person and remote assessment scores by group.

Notes: No between group differences were identified in ICC of in-person and remote assessments at any timepoint. Abbreviations- 5xSTS; time taken to perform five repeated sit-to-stands at maximum speed, 60sSTS; number of complete sit-to-stand repetitions performed in 60 seconds.
